# Epidemiology of pediatric road traffic injuries: a multicenter hospital-based study in Ghana

**DOI:** 10.1186/s40621-025-00646-1

**Published:** 2025-12-01

**Authors:** Anthony Baffour Appiah, Michael Lowery Wilson, Peter Dambach, Mahsa MohammadNamdar, Alexis Dun Bo-ib Buunaaim, John Abanga Alatiiga, Vincent Ativor, Peter Donkor, Charles Mock

**Affiliations:** 1 Heidelberg Institute of Global Health, University of Heidelberg, Im Neuenheimer Feld 130.3, Heidelberg, 69120 Germany; 2 Section for Oral Health, Heidelberg Institute of Global Health, Heidelberg University, Heidelberg, Germany; 3 Department of Clinical Neurosciences, Injury Epidemiology and Prevention Research Group, Turku Brain Injury Centre, Turku University Hospital and University of Turku, Turku, Finland; 4 Department of Surgery, University for Development Studies, Tamale, Ghana; 5 Department of Trauma Orthopaedics, Tamale Teaching Hospital, Tamale, Ghana; 6 Department of Orthopedic and Trauma Surgery, Komfo Anokye Teaching Hospital, Kumasi, Ghana; 7 Department of Surgery, School of Medical Science, Kwame Nkrumah University of Science and Technology, Kumasi, Ghana; 8 Harborview Injury Prevention and Research Center, Harborview Medical Center, Seattle, USA

**Keywords:** Children, Adolescent, Traumatic brain injury, Road traffic accident, Geographic information system, Ghana

## Abstract

**Background:**

Road traffic injury (RTI) is a major threat to children and adolescents worldwide. RTIs account for 25.7 deaths per 100,000 people in the general population. Unlike in other western countries where road fatalities are declining, deaths in Ghana continue to rise. This study examined the injury characteristics and spatiotemporal patterns of pediatric RTI cases and inpatient fatalities across three zones in Ghana.

**Methods:**

This study employed a retrospective cross-sectional design, analyzing pediatric RTI data from three teaching hospitals in Ghana, with each hospital located in one of Ghana’s three geographic zones: northern, middle, and coastal/southern. The study included all pediatric RTI cases captured between 2021 and 2024. Data on sociodemographic, spatial-temporal information, type of injury, injury severity, and admission outcome were analyzed. Descriptive statistics and Chi-square tests were used to compare groups at *p* < 0.05. Quantum Geographic Information System (QGIS) was used to develop choropleth maps.

**Results:**

A total of 1,485 pediatric RTI cases were included. Boys constituted 72.3%. Adolescents aged 13–18 years (45.6%) and school children aged 6–12 years (32.4%) were the most affected age groups. The leading causes of RTI were pedestrian knockdown (51.1%) and motorcycle crash (33.2%). While pedestrian knockdowns were widespread across the country, motorcycle crashes were dominant in the northern zone. Head injury was commonly reported among patients seen in the middle (60.4%) and northern (59.5%) zones, while lower limb injuries (54.3%) were most frequently seen in the southern zone. Mortality rates differed among the zones: 6.9% northern, 2.8% southern, and 0% middle (*p* < 0.001).

**Conclusion:**

The differences in injury patterns, mortality rates, and crash types underscore regional disparities in risk exposure and point to the limited effectiveness of road safety interventions across the country. The local road safety authorities should intensify road safety education and law enforcement, with clear outcome indicators to monitor impacts. Improvements in road infrastructure are also necessary, which provide separate routes for pedestrians with strict adherence.

**Supplementary Information:**

The online version contains supplementary material available at 10.1186/s40621-025-00646-1.

## Background

In Ghana, most schoolchildren commute on foot, while others commonly travel by motorcycle, public transport, school buses, or, in some cases, private vehicles [1]. However, the choice of transportation mode is influenced by multiple factors, including availability, cost, urgency of situation, distance to destination, household socio-economic status, and importantly the child’s geographical location [2, 3]. These are systemic and structural challenges that require broad stakeholder engagement and a sustained, long-term approach for effective resolution. However, in the short term, there is a pressing need for targeted interventions to promote children’s safety and safer road use. These measures should be evidence-based and context-specific, aimed at complementing and strengthening existing road safety strategies across Ghana.

More than 1.19 million people die each year as a result of road traffic injuries (RTI), with low- and middle-income countries accounting for 90% of these deaths [2, 4]. RTIs are the tenth leading cause of years lived with disability among pediatric population [2, 4]. In Sub-Saharan Africa, RTIs account for 17.6 deaths per 100,000 children under 19 years each year, while Ghana records an average of 25.7 traffic-related deaths per 100,000 population [5]. Unlike other regions where road fatalities are declining, deaths in Ghana continue to rise [5, 6].

A Delphi study conducted to generate consensus on road safety priorities in Ghana identified unattended vehicles along roadways, two- and three-wheeled motorcycles, distracted driving, speeding and driving skills as key contributors to the high burden of RTI [7]. However, peculiar characteristics of pediatric RTIs were not discussed, despite acknowledging the vulnerability of young people. Guerrero et. al [8] in a population-based study in Ghana’s capital found that pediatric RTIs were associated with bicycle and mini-bus use. However, none of circumstances of injury examined such as playing, walking to or from school, riding to or from work, hawking, etc., was statistically significant with pediatric RTIs in under 15 years [8]. Another study by Baffour Appiah et al. reported that non-helmeted motorcycle users under 25 years of age were nine folds more likely to sustain head injuries compared helmeted users in northern Ghana [9]. However, other important pediatric road user groups were not included, and the single-center design limits the generalizability of study findings to reflect the true injury and risk patterns across Ghana.

As traffic density and the nature of the road network vary across the country, it is expected that the risk profile of pediatric RTI and deaths in Ghana varies by region and zone [10, 11]. Therefore, this study sought to analyze the risk profile of road traffic injuries among children across the three main ecological zones in Ghana using data from a multi-center hospital-based study. The core principles of descriptive epidemiology were applied to explore pediatric RTI cases by person, place, and time, with the aim of informing region and zone-specific contextual interventions.

## Patients and methods

### Study design and study population

This study employed a quantitative, retrospective cross-sectional design, using secondary data on pediatric RTI patients reported at three teaching hospitals in Ghana. Eligible patients met the following inclusion criteria: (i) age below 19 years, (ii) involvement in road traffic–related injuries or deaths, (iii) occurrence of the injury or death within Ghana, and (iv) presentation for treatment at one of the study centers between January 2021 and September 2024.

### Study setting

The study was conducted in three teaching hospitals in Ghana. Ghana has sixteen administrative regions, which are grouped into three main ecological zones: the Coastal (or Southern) Zone, the Middle Zone, and the Northern-Savannah Zone. Regions in the Northern Zone are less resourced, with poorer road and social infrastructures and limited modes of transport compared to the Middle and Southern Zones [12, 13]. The study employed two-stage sampling to select the teaching hospitals and study participants. Since the Northern Savannah Zone hosts only one teaching hospital, it was automatically included. For the Middle and Coastal Zones, one teaching hospital was randomly selected from the list of hospitals in each zone. The facilities included in the study were Tamale Teaching Hospital (TTH) in the Northern Zone, Komfo Anokye Teaching Hospital (KATH) in the Middle Zone, and Cape Coast Teaching Hospital (CCTH) in the Southern Zone. The study included all pediatric RTI patients treated at each selected hospital. Due to the limited number of tertiary hospitals, these teaching hospitals are strategically positioned to train healthcare professionals and to manage major trauma and complex medical cases across in Ghana’s regions and districts [14–16].

### Data sources and acquisition

This study was part of a broader pediatric road traffic injury project in Ghana, a pilot multi-center trauma registry. The registry data were collected through review of medical records and administration of questionnaires between January 2021 and September 2024. Two trained Trauma Registrars (TRs) per center conducted the review of patients records from the Electronic Medical Records at each the study centers, using the WHO International Classification of Diseases 10th Revision (ICD-10) as a guide [17]. All review and extraction conducted before September 2023 relied exclusively on electronic medical records. However, during the data collection period, additional data were obtained through the review of paper-based medical records at the emergency room and wards to complement the electronic records of pediatric patients admitted during that period. The review focused on all injuries related to road transport accidents in patients under 19 years in accordance with the list of ICD-10 codes (Appendix Table 1 A). The system captured patient sociodemographic characteristics, spatial and temporal information, type of road users, mechanism of injury, injury.

During the prospective data collection, the TRs approached every young injured patient reported at the Accident and Emergency unit to verified their age and causes of injury. This was followed by written informed consent from each patient aged 16 years and above, and from parents or guardian for minors under 16 years, with assent obtained from the minor themselves. Each participant was assisted by the TRs in completing the questionnaire on patient and crash characteristics.

**Fig. 1:**
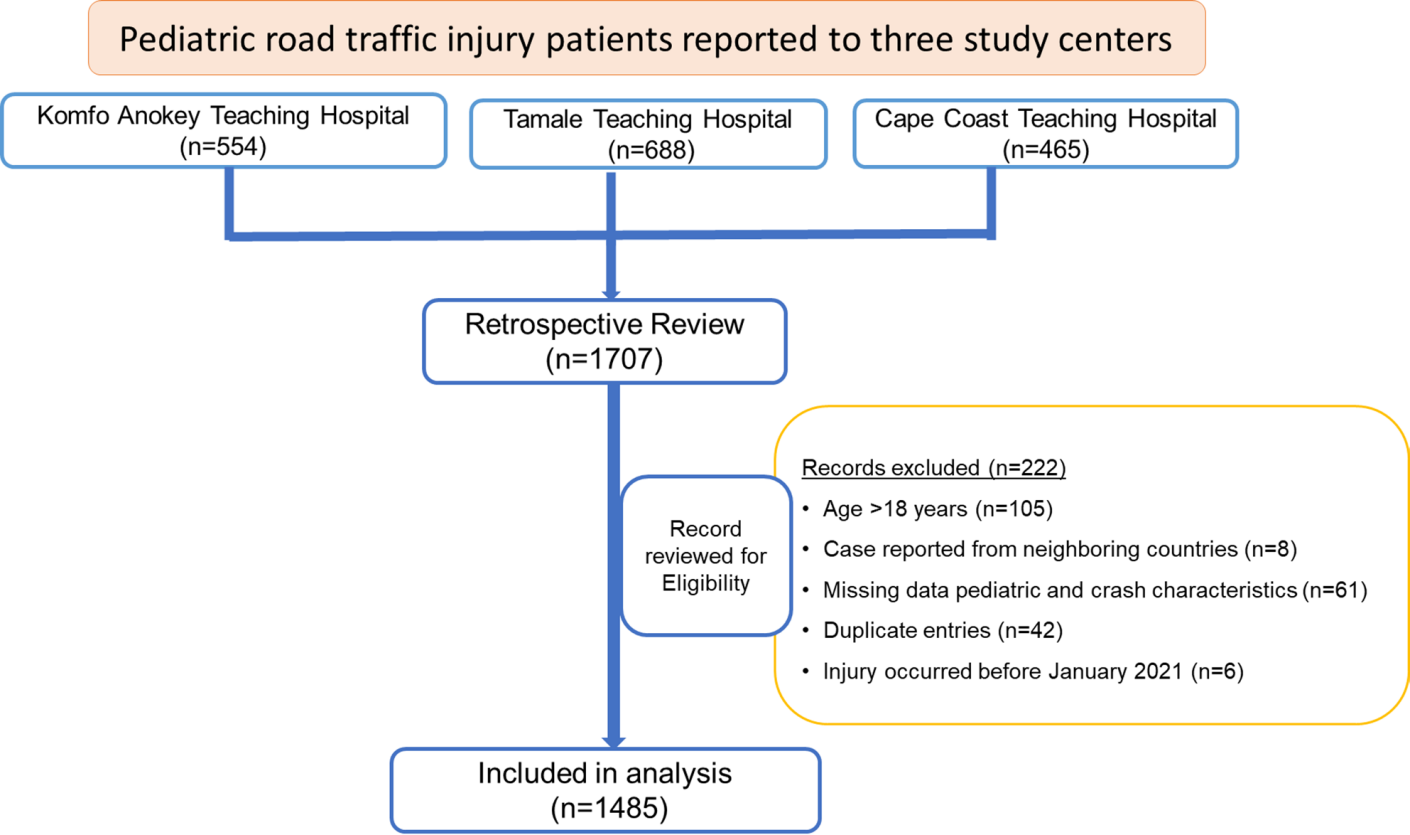
Flowchart of medical record review at the three study centers in Ghana, January 2021–September 2024

Daily data entry was performed using KoboToolbox, an electronic mobile data collection application. A double-validation approach was employed to identify and correct discrepancies and missing data during the data collection. This process included biweekly virtual validation meetings to review entries. The TR addressed identified errors under the guidance of a consultant trauma surgeon, who served as the site collaborator. Once resolved, the TR notified the lead investigator, who verified the changes and marked the entries as complete. The final analysis included 1,485 pediatric patients aged less than 19 years who met the eligibility criteria as illustrated in the flowchart (Fig. 1).

### Study variables

The study included all pediatric traffic-related injuries and in-patient deaths in accordance with the ICD-10. The spatial comparation were made using two variables; the hospital patient received treatment and the region where the injury occurred. Variables under the person domain included age, sex, mechanism of injury, child’s activity at crash, type of injuries, severity of injury, and inpatient status at end of study. Variables under the place domain included the region and community (rural, peri-urban or urban) where injury occurred were obtained from the patients’ crack and injury history note. Lastly, temporal variables were time of the day, day of the week, month, season injury occurred, and year of injury occurrence. The major seasons covered included rainy season (May to October) and dry season (November to April). The Harmattan season (December to February) was excluded from the dry season due to its peculiar significance in influencing the risk of road accidents.

### Data analysis

The quantitative data was analyzed using Statistical Package of Social Sciences (SPSS) version 22. Descriptive statistics such as frequencies, percentages, and means (standard deviation, SD) were used to summarize data. The difference in proportions between the three teaching hospitals was assessed with the Pearson’s Chi-square test and Fisher’s Exact test (when the expected count of a cell was < 5) at *p* < 0.05. The Spatial analysis was conducted using the Quantum Geographic Information System (QGIS) software, version 3.40.6. The georeferenced database of the new 16 Administrative Regions in Ghana is freely available online in SHP format (shapefile), USAID Ghana HPNO [18]. Choropleth maps were generated to compare the distribution of pediatric RTI cases and mortality for each region. Data was presented using tables, figures, and maps.

## Results

### Pediatric characteristics

Between 2021 and 2024, a total of 1,485 pediatric RTI cases were included in the study. The mean age was 10.8 (± 5.4). The most affected demographic groups were adolescents aged 13–18 years (45.9%) and boys (72.2%) (Table 1). The overall in-hospital mortality rate was 3.6% (54/1,485), with a higher proportion of deaths occurring among adolescents (51.9%, 28/54) and males (70.4%, 38/54).

**Table 1:** Crash characteristics of pediatric road traffic injuries, Ghana, 2021–2024

Variable	All centers *N* = 1485(%)	KATH *n* = 505(%)	TTH *n* = 654(%)	CCTH *n* = 326(%)	*P*-value
**Age of pediatric**					0.023
Infants/Toddler(0-2yrs)	92 (6.2)	21 (4.2)	52 (8.0)	19 (5.8)	
Preschool (3-5yrs)	240 (16.2)	70 (13.9)	115 (17.6)	55 (16.9)	
School-age child (6–12 yrs)	472 (31.8)	155 (30.7)	209 (32.0)	108 (33.1)	
Adolescent (13–18 yrs)	681 (45.9)	259 (51.3)	278 (42.5)	144 (44.2)	
**Sex of pediatric**					0.055
Male	1072 (72.2)	366 (72.5)	487 (74.5)	219 (67.2)	
Female	413 (27.8)	139 (27.5)	167 (25.5)	107 (32.8)	
**Type of road user**					< 0.001
Pedestrian	759 (51.1)	300 (59.4)	259 (39.6)	200 (61.3)	
Motorcyclist	493 (33.2)	138 (27.3)	304 (46.5)	51 (15.6)	
Tricyclists	107 (7.2)	5 (1.0)	63 (9.6)	39 (12.0)	
Bus occupant	83 (5.6)	47 (9.3)	21 (3.2)	15 (4.6)	
Car occupant	43 (2.9)	15 (3.0)	7 (1.1)	21 (6.4)	
**Pediatric situations at crash**					< 0.001#
On an errand	340 (22.9)	55 (10.9)	162 (24.8)	123 (37.7)	
Going to School	387 (26.1)	191 (37.8)	153 (23.4)	43 (13.2)	
Playing along roadside	157 (10.6)	34 (6.7)	65 (9.9)	58 (17.8)	
Travelling (with/out parent)	315 (21.2)	67 (13.3)	213 (32.6)	35 (10.7)	
Going to work	105 (7.1)	54 (10.7)	17 (2.6)	34 (10.4)	
Hawking along the roadside^a^	15 (1.0)	6 (1.2)	9 (1.4)	0 (0.0)	
Going to place of worship	62 (4.2)	31 (6.1)	10 (1.5)	21 (6.4)	
Not specified	104 (7.0)	67 (13.3)	64 (3.8)	12 (3.7)	
**Remoteness Community**					< 0.001#
Rural	482 (32.5)	202 (40.0)	175 (26.8)	105 (32.2)	
Peri-urban	429 (28.9)	66 (13.1)	145(22.2)	218 (66.9)	
Urban	574 (38.7)	237 (46.9)	334 (51.1)	3 (0.9)	
**Season injury occurred**					0.006
Dry season (Nov. Mar.-Apr.)	410 (27.6)	142 (28.1)	175 (26.8)	93 (28.5)	
Rainy season (May - Oct.)	714 (48.1)	263 (52.1)	290 (44.3)	161 (49.4)	
Harmattan season (Dec. - Feb.)	361 (24.3)	100 (19.8)	189 (28.9)	72 (22.1)	
**Time injury occurred**					< 0.001
Morning (06:00–11:59)	435 (29.3)	192 (38.0)	140 (21.4)	103 (31.6)	
Afternoon (12:00–17:59)	532 (35.8)	192 (38.0)	222 (33.9)	118 (36.2)	
Evening (18:00–20:59)	347 (23.4)	75 (14.9)	197 (30.1)	75 (23.0)	
Night (21:00–05:59)	171 (11.5)	46 (9.1)	95 (14.5)	30 (9.2)	

#p-value from Fisher’s Exact test; TTH: Tamale Teaching Hospital, KATH: Komfo Anokey Teaching Hospital; CCTH: Cape Coast Teaching Hospital; a- this refers to children selling goods while walking along the roadside

### Crash characteristics of pediatric road traffic injuries

**Fig. 2:**
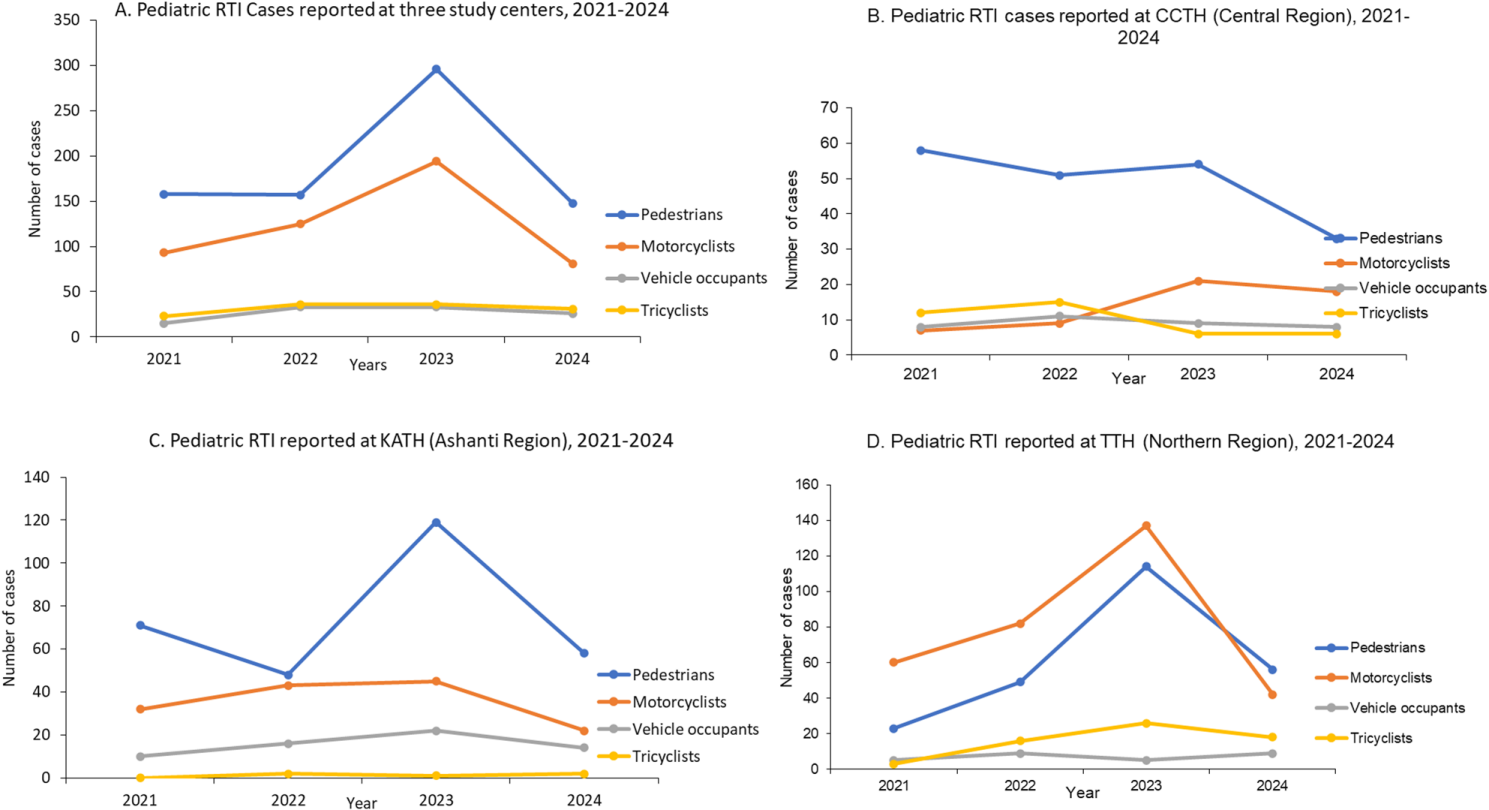
Trends in pediatric road traffic injury (RTI) patients by road user type and study center, 2021–2024. Trend analysis includes; (**A**) All centers combined; (**B**) Cape Coast Teaching Hospital (CCTH) in Central Region; (**C**) Komfo Anokye Teaching Hospital (KATH) in Ashanti Region; (**D**) Tamale Teaching Hospital (TTH) in Northern Region

RTI occurred most frequently during school trips (26.1%), running errands (22.9%), and while travelling (21.2%). The child’s activity before the accident varied significantly across the study centers (*p* < 0.001) (Table 1). The top two leading causes of RTI were pedestrian knockdown (51.1%) and motorcycle crash (33.2%). While pedestrian crashes were very common among patients reported from Ashanti (33.2%), Northern (25.6%) and Central (24.9%). More than 50% of patients involved in motorcycle crashes were reported from Northern, with 21.1% from Ashanti and only 10.5% from Central Regions (Table 2). Similar pattern was observed in subgroup trend analysis of injury and inpatient mortality by road user type across the three study centers (Fig. 2).

**Table 2:** Type of road users involved by region pediatric road traffic injury occurred, Ghana, 2021–2024

Region by zone	Pedestrian ***n = 756(%)***	Motorcyclist ***n = 493 (%)***	Tricyclists ***n = 107 (%)***	Bus occupant ***n = 83(%)***	Car occupant ***n = 43 (%)***	All ***n = 1485 (%)***
Northern zone						
North East	9 (1.2)	18 (3.7)	0 (0.0)	5 (6.0)	1(2.3)	33 (2.2)
**Northern**	194 (25.6)	250 (50.7)	51 (47.7)	13 (15.7)	5 (11.6)	513 (34.5)
Savannah	24 (3.2)	11 (2.2)	6 (5.6)	1 (1.2)	1(2.3)	43 (2.9)
Upper East	19 (2.5)	16 (3.2)	4 (3.7)	1 (1.2)	0 (0.0)	40 (2.7)
Upper West	3 (0.4)	2 (0.4)	0 (0.0)	0 (0.0)	0 (0.0)	5 (0.3)
Middle zone						
Ahafo	5 (0.7)	2 (0.4)	0 (0.0)	1 (1.2)	1 (2.3)	9 (0.6)
**Ashanti**	252 (33.2)	104 (21.1)	5 (4.7)	37 (44.6)	14 (32.6)	412 (27.7)
Bono	5 (0.7)	7 (1.4)	0 (0.0)	1 (1.2)	0 (0.0)	13 (0.9)
Bono East	10 (1.3)	7 (1.4)	1 (0.9)	1 (1.2)	0 (0.0)	19 (1.3)
Eastern	10 (1.3)	4 (0.8)	0 (0.0)	4 (4.8)	0 (0.0)	18 (1.2)
Oti	4 (0.5)	6 (1.2)	1 (0.9)	1 (1.2)	0 (0.0)	12 (0.8)
Southern/Costal zone						
**Central**	189 (24.9)	52 (10.5)	34 (31.8)	9 (10.8)	16 (37.2)	300 (20.2)
Greater Accra	1 (0.1)	0 (0.0)	0 (0.0)	0 (0.0)	0 (0.0)	1 (0.1)
Volta	0 (0.0)	0 (0.0)	0 (0.0)	0 (0.0)	0 (0.0)	0 (0.0)
Western	27 (3.6)	11 (2.2)	5 (4.7)	9 (10.8)	5 (11.6)	57 (3.8)
Western North	7 (0.9)	3 (0.6)	0 (0.0)	0 (0.0)	0 (0.0)	10 (0.7)

Bold text indicates the regions hosting the three teaching hospitals that served as study centers: Northern Region (TTH), Ashanti Region (KATH), and Central Region (CCTH), with Tamale, Kumasi, and Cape Coast as their respective capital cities

### Temporal pattern of pediatric road traffic injuries

**Fig. 3:**
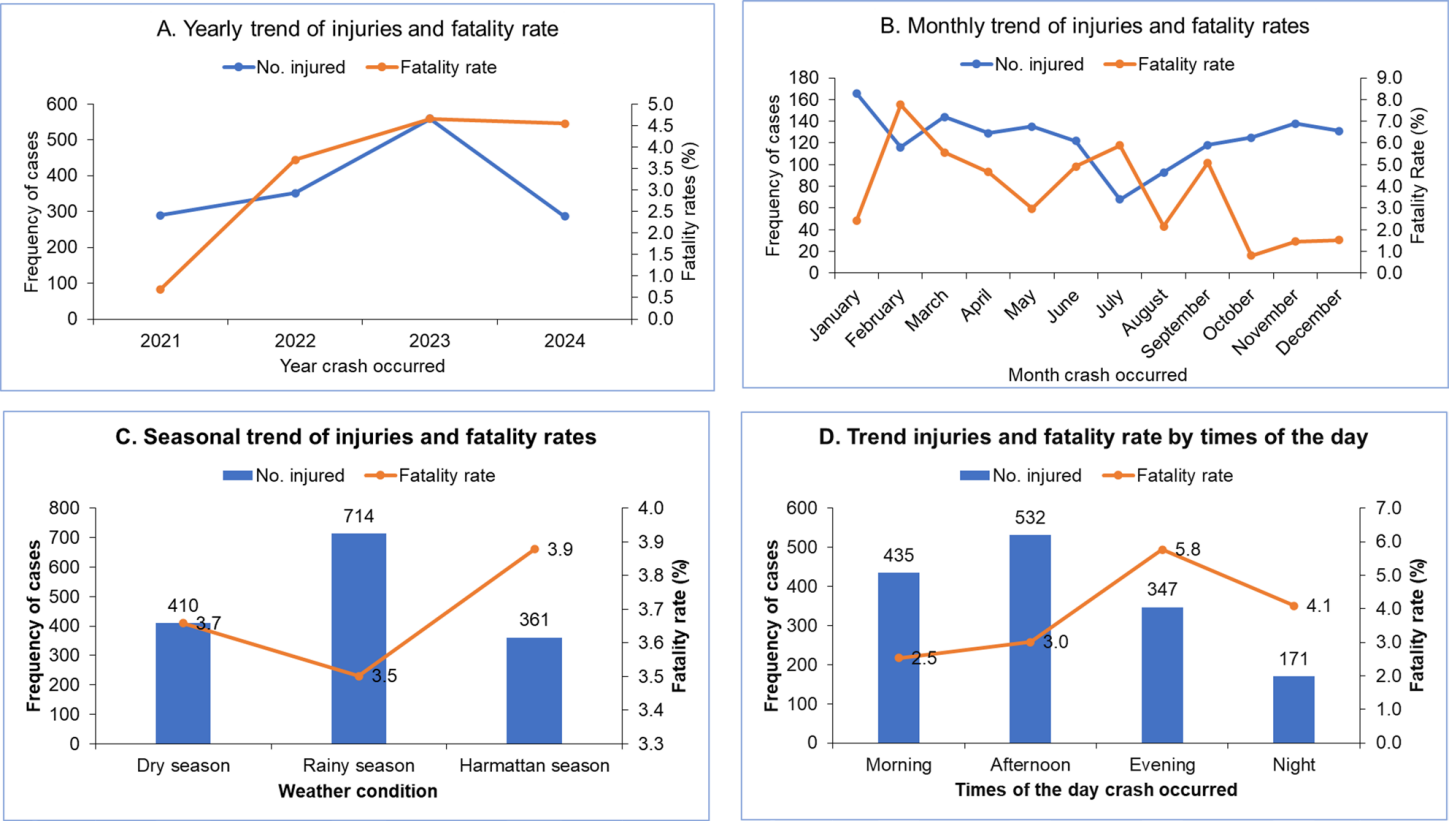
Temporal patterns of pediatric road traffic injuries and inpatient mortality rates across three study centers in Ghana, January 2021–September 2024. Subgroup analyses include: (**A**) annual trends, (**B**) monthly distribution, (**C**) seasonal variation, and (**D**) patterns by time of day

Overall, there was a steady year-on-year increase in the number of cases, ending in a peak in 2023. Data were only gathered for 9 months in 2024. A similar trend was observed in the fatality rate, with an overall increase leading up to a peak (Fig. 3A). Monthly trends showed irregular fluctuations in both pediatric RTI cases and fatality rates over the study period (Fig. 3B). Nearly half (48.1%) of reported pediatric RTIs occurred during the rainy season and differed significantly across the three-study centers (*p* = 0.006). The rate of RTI was high in the afternoon (35.8%) and morning (29.3%) and similar disparity was seen at the TTH and CCTH, but equal rate for both periods at KATH (*p* < 0.001) (Table 1). However, fatality rate was higher during Harmattan season (Fig. 3C) and evenings of the day (Fig. 3D).

### Spatial pattern of pediatric road traffic injuries and mortality

**Fig. 4:**
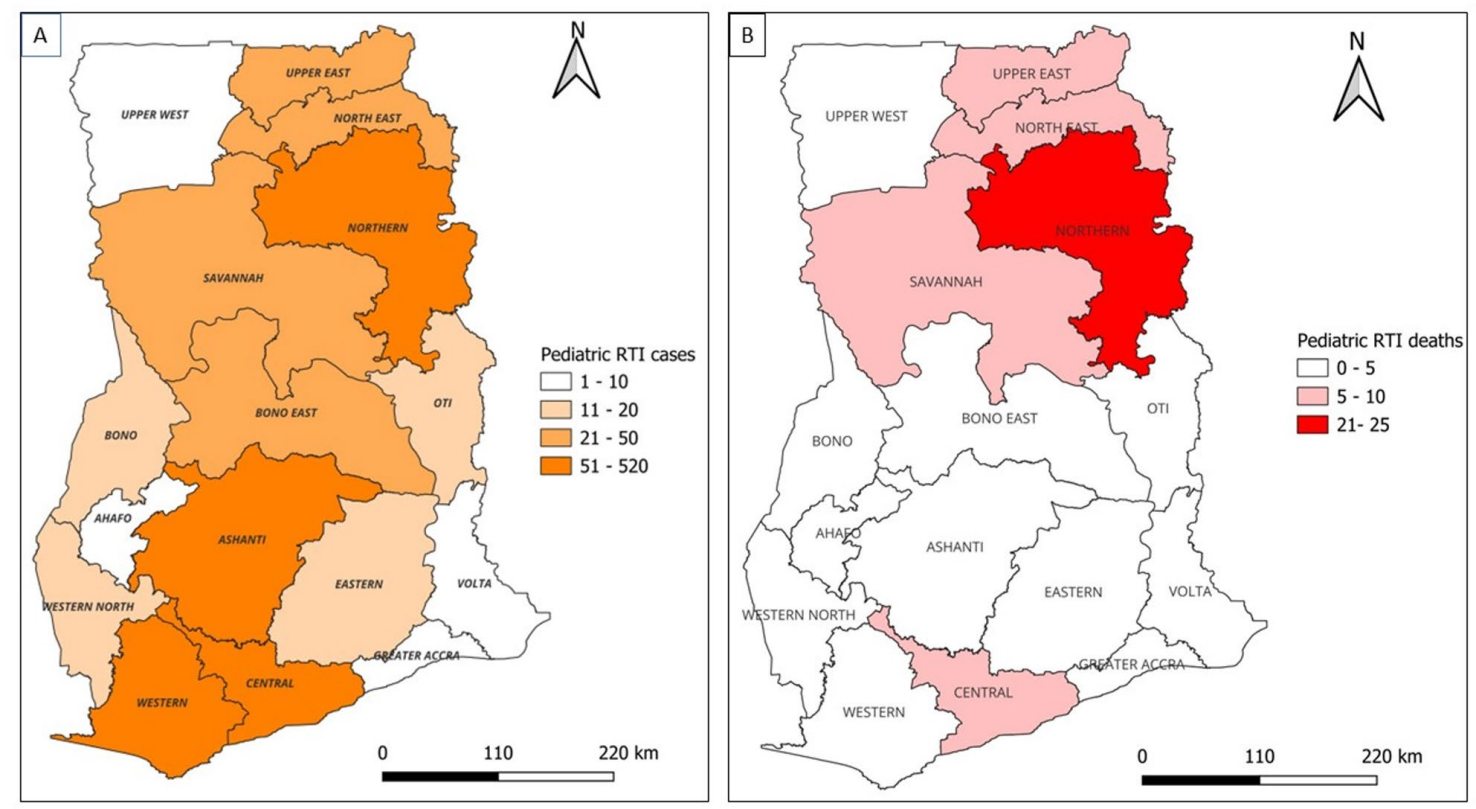
Geospatial distribution of pediatric road traffic injury (**A**) and in-patient deaths (**B**) presented at three Teaching hospitals in Ghana, January 2021– September 2024

Over one-third of pediatric RTI cases were reported from urban (38.7%) and rural (32.5%) communities, which differ significantly across the three hospitals(*p* < 0.001) (Table 1). Regional differences in pediatric RTI and inpatient mortality between urban and rural areas are shown in Appendix Table A2. As shown in Fig. 4, the majority of pediatric RTI cases originated from regions in which the study hospitals are situated, with 490 (34.2%) cases from the Northern Region, 412 (28.8%) cases from the Ashanti Region and 292 (20.4%) cases from the Central Region. Moreover, nearly half (42.6%) of pediatric RTI deaths were reported from the Northern Region, 14% (8 cases) from the Central region, and seven cases each from the Savannah and Upper East Regions. However, no death was reported from the Ashanti Region.

### Pattern and outcome of pediatric road traffic injuries

**Fig. 5:**
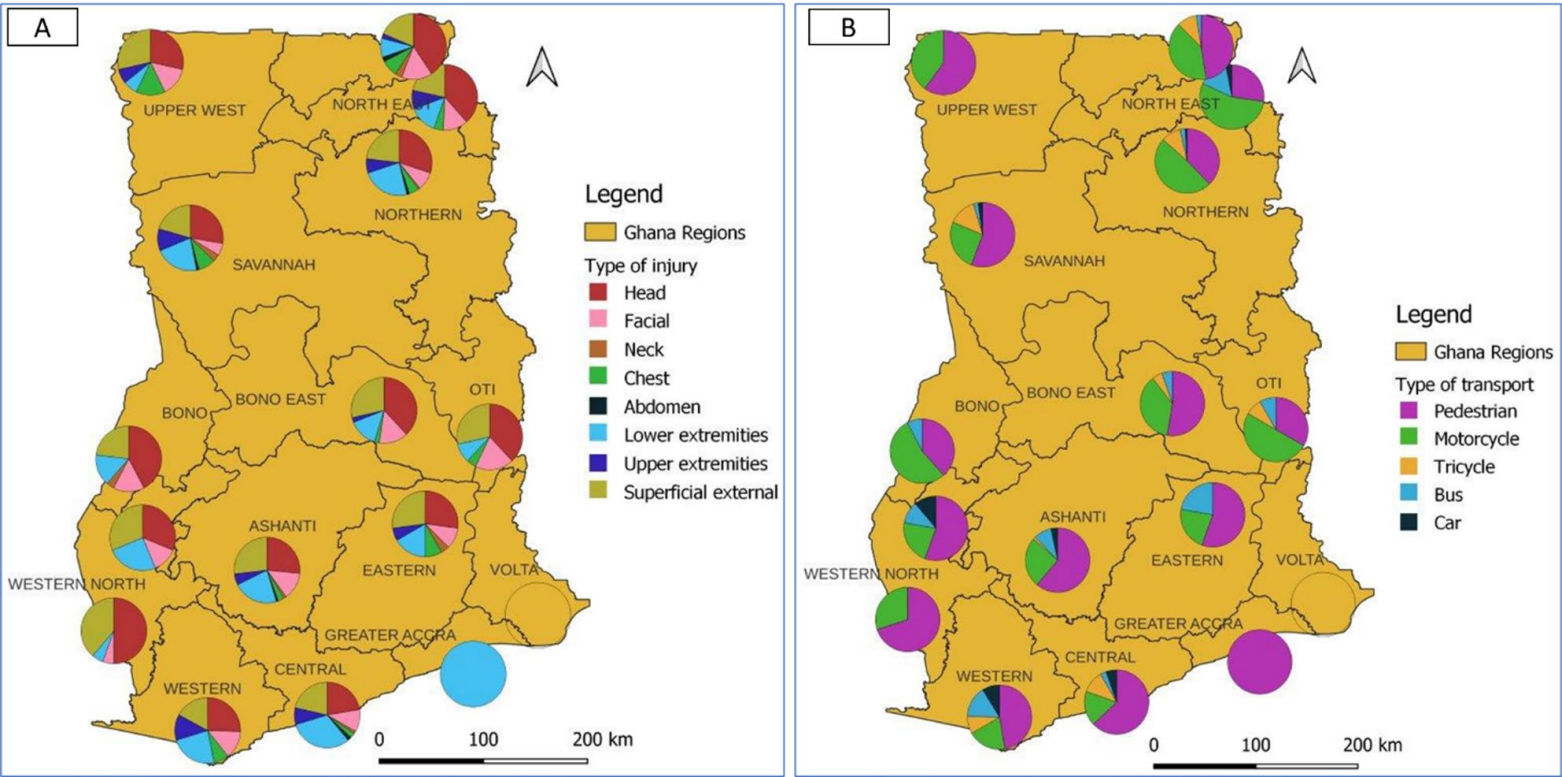
Geospatial distribution of (**A**) type of injuries and (**B**) causes of pediatric road traffic injuries by Regions, Ghana, 2021–2024

Common anatomical injuries reported were head injuries (54.9%), lower limbs (45.5%) and superficial injuries (46.4%). While head injuries were predominant at KATH (60.4%) and TTH (59.5%), lower limbs injuries were the most recorded at CCTH (54.3%) (Table 3). Similarly, the rate of head injuries was high in regions within Northern and Middle Zones, whereas lower extremity injuries were more common in the Southern Zone (Fig. 5). The severity of pediatric RTI cases differs significantly among the three study hospitals (*p* < 0.005). A much higher rate of severe injuries (ISS > 15) was observed at KATH (28.9%) compared to 16.7% at TTH and 15.1% at CCTH. Similarly, by study center, TTH recorded the highest rate of inpatient mortality (6.9%) compared to 2.8% at CCTH and none at KATH (*p* < 0.005) (Table 3).

**Table 3:** Injury characteristics of pediatric road traffic injury, Ghana, 2021–2024

Variable	All centers *N* = 1485(%)	KATH *n* = 505(%)	TTH *n* = 654(%)	CCTH *n* = 326(%)	*P*-value
**Anatomical injury**					
Head injury	816 (54.9)	305 (60.4)	389 (59.5)	122 (37.4)	< 0.001
Facial injury	327 (22.0)	145 (28.7)	122 (18.7)	60 (18.4)	< 0.001
Neck injury	35 (2.4)	15 (3.0)	13 (2.0)	7 (2.1)	0.529
Spinal injury	14 (0.9)	7 (1.3)	6 (0.9)	1 (0.3)	0.289
Chest injury	106 (7.1)	26 (5.1)	62 (9.5)	18 (5.5)	0.008
Abdominal injury	34 (2.3)	10 (2.0)	15 (2.3)	9 (2.8)	0.764
Upper limb injury	194(13.1)	56 (11.1)	86 (13.1)	52 (16.0)	0.127
Lower limb injury	675 (45.5)	231 (45.7)	267 (40.8)	177 (54.3)	< 0.001
Superficial injury	689 (46.4)	294 (58.2)	277 (42.4)	118 (36.2)	< 0.001
**Injury severity level**					< 0.001
Mild (< = 8)	857 (57.7)	283 (56.0)	384 (58.7)	190 (58.3)	
Moderate (9–15)	324 (21.8)	76 (15.0)	161(24.6)	87 (26.7)	
Severe (16–24)	228 (15.4)	112 (22.2)	77 (11.8)	39 (12.0)	
Critical (> = 25)	76 (5.1)	34 (6.7)	32 (4.9)	10 (3.1)	
**Deposition of patient**					< 0.001#
Discharged home	753 (50.7)	437 (86.5)	33 (5.0)	283 (86.8)	
Still on admission	557 (37.5)	50 (9.9)	500 (76.5)	7 (2.1)	
DAMA	121 (8.1)	18 (3.6)	76 (11.6)	27 (8.3)	
Mortuary	54 (3.6)	0 (0.0)	45 (6.9)	9 (2.8)	
**In-hospital mortality**					< 0.001#
Died	54 (3.6)	0 (0.0)	45 (6.9)	9 (2.8)	
Survived	1431 (96.4)	505 (100.0)	609 (93.1)	317 (97.2)	

DAMA: Discharged against medical advice, **#**p-value from Fisher’s Exact test, TTH: Tamale Teaching Hospital, KATH: Komfo Anokey Teaching Hospital; CCTH: Cape Coast Teaching Hospital

## Discussion

This paper provides critical evidence to inform policy decisions and the implementation of targeted road safety interventions in the three study regions and zones in Ghana. The analysis revealed an age-related increase in the trend of RTI and mortality, with the highest burden among male adolescents aged 13 to 18 years and in the Northern Region. Head injury was commonly reported among patients seen at KATH (60.4%) and TTH (59.5%), while lower limb injuries were most seen at CCTH. The leading causes of RTIs were pedestrian knockdown (51.1%) and motorcycle crash (33.2%). While pedestrian knockdowns were widespread across the country, motorcycle crashes were dominant in the five Northern Regions. Child’s activity associated with pediatric road traffic crashes differed significantly across the three study centers.

We found that adolescents (13–18 years) experienced a higher rate of injury than children under 13 years. This is comparable with findings from Albargi et al., who noted high injury rates in schoolchildren (37.8%) and adolescents (31.8%) [19], Furthermore, Hartka et al. reported that the 15–18 age group accounted for over 50.9% of injuries [20]. The age pattern of RTI observed in this study could be explained by adolescents’ heightened exposure to risk-related behaviors, which may result from their growing independence and increased mobility relative to children [3, 6]. This underscores the need for age-targeted road safety interventions to reduce the burden of adolescent and childhood RTIs. Our findings that boys were the most affected by RTIs were consistent with existing literature, which reported a higher incidence of RTIs and deaths among male road users [6, 21, 22].

The study revealed proportion of RTI in boy, with an average of 72.2% across various centers. This is comparable with 62.8% and 67.2% in Ghana [8, 23]. Boys have greater exposure to traffic due to their greater in involvement in outdoor games and recreational activities that involved traveling at longer distance using roadways [21]. Usually through cycling, walking at earlier ages, and for longer durations than girls. This potentially increases their physical exposure to varied traffic environments which predisposes them to risk for traffic injuries. This is compounded by a higher propensity for risk-taking behaviors, including alcohol use before driving, speeding, overestimation of driving skills, lack of helmet and seatbelt use [6, 24].

The trend of pediatric RTI cases and mortality rates was comparable with the literature, which reported a similar trend in RTI in Ghana [25] and worldwide [2]. The upward trend may reflect increased exposure to traffic hazards, improved reporting, or higher referral rates during the early years [25]. However, the sharp decline observed in 2024 deviates from the overall trend. This is partly due to the early conclusion of data collection, as injuries occurring between October and December 2024 were not captured. However, injury and fatality rates remained high consistent with reported pattern of RTI-related deaths in Ghana [26, 27]. This highlights the need to strengthen road safety campaigns, stricter enforcement of traffic regulations and improve road infrastructure, particularly pedestrian facilities.

We observed an irregular monthly pattern of pediatric RTI cases and fatalities over the study period, with most cases reported during the first and fourth quarters of the years. These findings corroborate previous crash analyses in Ghana, which showed that over 32% of all traffic accidents occurred in the fourth quarter [26]. Also, the rate of RTI was higher in the afternoon and morning, consistent with Seid et al. [28], who reported a greater incidence of traffic injuries in the afternoon compared to the morning. This reflects the temporal influence of different social events and weather conditions on pediatric road traffic crashes. The peaks in the first and fourth quarters could be linked to the Christmas and New Year seasons and related activities, which influence economic activities, traffic volume, and travel. Moreover, the substantial proportion of injuries and deaths occurring during the rainy season is consistent with findings from previous studies [12]. Finding could be attributed to impaired road visibility and slippery road for road users, increasing the likelihood of vehicles skidding or veering off the road.

The findings indicate that pediatric RTI cases were predominantly reported from regions hosting the study hospitals, notably 34.2% from the Northern Region, 28.8% from Ashanti, and 20.4% from the Central Region. These regional variations likely reflect the differences in the access and capacity of healthcare facilities, risk exposure pattern and population density across the ecological zones [2]. For instance, in terms of healthcare access, the Northern Zone has only one teaching hospital, compared to two each in the Southern and Middle Zones. This partly explains the higher referral rate of pediatric RTI cases to TTH compared to KATH and CCTH. However, it is possible that not all pediatric RTI cases were transferred to or presented at the study centers, potentially leading to an underrepresentation of the true burden of pediatric RTI in study centres. Moreover, the existing surveillance systems, such as those by the Building and Road Research Institute (BRRI) and the District Health Information Management System 2 (DHIMS-2) that primarily capture crash data have limited detail on injury characteristics [12, 25]. Underreporting or failure to present to healthcare facilities may have contributed to cases not being captured in this study [25].

In this study, RTI-related inpatient mortality rates were relatively low across the study sites; this is comparable with national traffic mortality estimates [26]. Our overall inpatient mortality rate (3.6%) was comparable to the 4% reported in Uganda, but less than half the 7.7% rate reported in Saudi Arabia [19, 29]. This discrepancy may be attributed to differences in patient characteristics, such as a varying focus on traumatic brain injuries. Inpatient mortality from RTIs has been associated with delays in identifying life-threatening injuries and in referring patients to higher-level trauma centers [30]. This could explain why nearly half of all reported pediatric RTI deaths occurred in the Northern Region. Further analysis of deaths recorded at TTH showed that relatively higher mortality rates among patients referred from rural and peri-urban communities in the Northern Region (Appendix Table 2 A**)**, where long travel distances, poor road conditions, and limited access to ambulances are common challenges. Unexpectedly, no inpatient injury-related deaths were reported at KATH, a finding that warrants further investigation. A plausible explanation may be the under-ascertainment of trauma-related deaths during data collection, or the possibility that severely injured patients who were transported died upon arrival and were transferred directly to the mortuary.

Pediatric patients were more likely to sustain traumatic brain injuries and lower extremity injuries, a pattern consistent studies on RTI and related deaths across the country [31, 32]. Nearly 33% of injured patients suffered head injury in Ghana [32]. The rate of head injuries was high in the Northern and Ashanti Regions, whereas lower extremity injuries were common in the Central Region. The elevated risk of TBI among children could be linked to high motorcycle dependency and a low rate of helmet use in the Ghanaian subpopulation [9, 33]. Turkson et al. [34] reported that the rate of riders not wearing helmets was higher in the northern (53.2%) compared to the southern (33%) zones of Ghana. Even among users, non-standard helmets are commonly used, which offer significantly less protection compared to certified standard helmets [35]. However, the choice of helmet type in Ghana is often influenced by availability and cost, offering opportunity for deliberate government policies and interventions [36]. Conversely, the high incidence of lower-extremity injuries is likely attributable to pedestrian and motor vehicle crashes, which was dominant in southern Ghana [27]. Aside poor road infrastructure [37], this situation is further worsened by limited adult supervision for school children, low awareness of road safety, and weak enforcement of speed limits. The variations in injury patterns and mechanisms underscore the contextual differences in traffic risk across Ghana, emphasizing the urgent need for targeted, evidence-based interventions to enhance pedestrian safety on roads and streets.

### Strength and limitations

This study provides valuable insights to inform more targeted contextual interventions to address pediatric road traffic crash exposures across Ghana. Unlike previous studies that rely on population-based crash data (e.g. from BRRI and DHIMS-2), which often lack detailed clinical information, or on household surveys prone to high recall bias, our study utilizes clinical data from hospital records. Another strength of the study is the application of spatial techniques which provided valuable insights into the geographic and temporal patterns of pediatric RTI cases in Ghana. Hence, to the best of our knowledge, this study is one of the first clinical study on pediatric RTI cases which use a representative data from major teaching hospitals from the three ecological zones in Ghana. However, our results are hospital-based, and this limits the generalization of the epidemiological findings. For instance, pre-hospital deaths and cases not reaching the teaching hospitals could lead to underestimation of injury and fatality. No inpatient deaths syndrome observed at KATH could be attributed to information bias arising from variations in death records, and limited access to critical care units, such as ICUs, where injury-related deaths are more likely to occur. Additionally, the potential inconsistencies in medical documentation and ICD-10 coding practices across hospitals limited the range of variables we were able to cover. Also, a cross-sectional study and use of pre-existing hospital data may introduce some selection bias, as children who did not present to the selected hospitals might differ from those included in this study.

### Policy implications

A sustained road safety awareness and education campaigns in communities targeting riders, pilons, drivers, and students in both Junior and senior high schools across Ghana. The programs should adopt simple, accessible and standardized training materials with local content. Also, the use of pre- and post-education evaluation with clear indicators at the individual and population levels will be essential in assessing effectiveness and guiding future interventions. Routine orientation of road safety promoters on the influence of climate change, weather conditions and social events on road safety will facilitate seamless dissemination to the public and vulnerable groups. These efforts should be accompanied by a sustained enforcement of road safety laws including helmet use and the provision of pedestrian road safety guides to assist children among others. Road design should align with international best practices by incorporating dedicated spaces for pedestrians, two-wheeled, and motor vehicles to improve safety and traffic flow. Addressing data issues requires a national injury surveillance system that links clinical data from teaching and peripheral hospitals across the country to provide more national clinical data on RTIs and deaths reported in Ghana.

## Conclusions

To conclude, our findings highlight a greater vulnerability of boys and adolescent to RTIs, and an observable increasing trend in pediatric RTI cases. A significant disparity was noted in inpatient mortality, with TTH in the Northern zone reporting higher rates than other centers in the Middle and Southern zones. Pediatric patients commonly presented with traumatic head injuries and lower extremity injuries, mirroring national patterns. Distinct geographical injury profiles emerged, showing a predominance of motorcycle-related injuries in the Northern zone, while pedestrian and motor vehicle-related injuries were more common in the Middle and Southern zones. Addressing these disparities requires the effective implementation of context-specific policy implications across all zones.

## Supplementary Information

Supplementary Material 1

## Data Availability

The anonymised data collected are available as open data via the OSF online data repository: [https://osf.io/r5fy7/?view_only=235b689570094e7687bd76a00adf2cc9].

## Abbreviations

BRRI: Building and Road Research Institute
CCTH: Cape Coast Teaching Hospital
DHIMS-2: District Health Information Management System 2
HPNO: Health, Population and Nutrition Office
KATH: Komfo Anokey Teaching Hospital
LMICs: Low- and Middle-Income Countries
MRH: Ministry of Roads and Highways
PRTI-GH: Pediatric Road Traffic Injury in Ghana
QGIS: Quantum Geographic Information System
RTI: Road Traffic Injury
TTH: Tamale Teaching Hospital
USAID: United States Agency for International Development
WHO: World Health Organization
YLD: Years lived with disability
