# Supplementary material for: Epidemiology of pediatric road traffic injuries: a multicenter hospital-based study in Ghana
Source: Inj Epidemiol. 2025 Dec 1;12:83. doi: 10.1186/s40621-025-00646-1 (PMC12690968; doi:10.1186/s40621-025-00646-1)
Supplement: Supplementary file 1 — Supplementary Material 1 [file 40621_2025_646_MOESM1_ESM.docx]

**Supplementary material**

Table 1A ICD-10 code recommended for circumstances of road traffic accidents and injuries

| ***A. Codes that describe the circumstances of the road traffic accident*** |
| --- |
| V01–V09: Pedestrian injured in transport accidents |
| *V01*: Pedestrian injured in collision with pedal cycle |
| *V02*: Pedestrian injured in collision with two- or three-wheeled motor vehicle |
| *V03*: Pedestrian injured in collision with car, pick-up truck, or van |
| *V09*: Other and unspecified transport accidents involving pedestrian |
| V10–V19: Pedal cyclist injured in transport accidents |
| V20–V29: Motorcycle rider injured in transport accidents |
| V30–V39: Occupant of three-wheeled motor vehicle |
| V40–V49: Occupant of car |
| V50–V59: Occupant of pick-up truck or van |
| V60–V69: Occupant of heavy transport vehicle |
| V70–V79: Bus occupant injured |
| V98–V99: Other and unspecified transport accidents |
| ***B. Codes describe the type of injury sustained in the road traffic accident*** |
| S00–S09: Head injuries (e.g., skull fractures, intracranial injuries) |
| S10–S19: Neck injuries |
| S20–S29: Thorax injuries |
| S30–S39: Abdomen, lower back, lumbar spine, pelvis injuries |
| S40–S99: Injuries to limbs (shoulder, arms, hip, legs, etc.) |
| T00–T14: Injuries involving multiple or unspecified body regions |
| T20–T32: Burns and corrosions (possible in vehicle fires after crashes) |

This table lists the ICD-10 codes that were used to obtain patients from the Electronic Medical Records (LHIMS) )[20].

**Table 2A Pattern of pediatric road traffic injury and deaths region and remoteness of community injury occurred, Ghana, 2021–2024**

| **Region by zone** | **Rural** | | **Peri-urban** | | **Urban** | | **All patients** | |
| --- | --- | --- | --- | --- | --- | --- | --- | --- |
|  | **Injury** | **deaths** | **Injury** | **deaths** | **Injury** | **deaths** | **Injury** | **deaths** |
| Ahafo | 8 | 0 | 0 | 0 | 1 | 0 | 9 | 0 |
| **Ashanti** | **121** | **0** | **63** | **0** | **228** | **0** | **412** | **0** |
| Bono | 9 | 0 | 2 | 0 | 2 | 0 | 13 | 0 |
| Bono East | 10 | 0 | 1 | 0 | 7 | 1 | 18 | 1 |
| **Central** | **101** | **4** | **3** | **0** | **188** | **4** | **292** | **8** |
| Eastern | 16 | 0 | 0 | 0 | 2 | 0 | 18 | 0 |
| Greater Accra | 0 | 0 | 0 | 0 | 1 | 0 | 1 | 0 |
| North East | 6 | 2 | 12 | 0 | 9 | 4 | 27 | 6 |
| **Northern** | **129** | **7** | **89** | **5** | **272** | **11** | **490** | **23** |
| Oti | 6 | 0 | 3 | 1 | 2 | 0 | 11 | 1 |
| Savannah | 11 | 2 | 19 | 5 | 6 | 0 | 36 | 7 |
| Upper East | 11 | 0 | 10 | 1 | 12 | 6 | 33 | 7 |
| Upper West | 1 | 0 | 0 | 0 | 4 | 0 | 5 | 0 |
| Western | 30 | 0 | 24 | 1 | 2 | 0 | 56 | 1 |
| Western North | 8 | 0 | 1 | 0 | 1 | 0 | 10 | 0 |

Bold text indicates the regions hosting the three teaching hospitals that served as study centers: Northern Region (TTH), Ashanti Region (KATH), and Central Region (CCTH), with Tamale, Kumasi, and Cape Coast as their respective capital cities.
